# Case report: Persistent shedding of a live vaccine-derived rubella virus in a young man with severe combined immunodeficiency and cutaneous granuloma

**DOI:** 10.3389/fimmu.2022.1075351

**Published:** 2022-12-08

**Authors:** Kimberly E. Bonner, Ellie Sukerman, Juventila Liko, Tatiana M. Lanzieri, Melissa Sutton, Emilio DeBess, Christopher Leesman, Joseph Icenogle, LiJuan Hao, Min-hsin Chen, Raeesa Faisthalab, Richard F. Leman, Paul R. Cieslak, Suk See DeRavin, Ludmila Perelygina

**Affiliations:** ^1^ Oregon Health Authority, Public Health Division, Portland, OR, United States; ^2^ Epidemic Intelligence Service, Center for Surveillance, Epidemiology and Laboratory Services, Centers for Disease Control and Prevention, Atlanta, GA, United States; ^3^ Division of Infectious Diseases, Oregon Health & Science University, Portland, OR, United States; ^4^ Division of Viral Diseases, Centers for Disease Control and Prevention, Atlanta, GA, United States; ^5^ Corvallis Family Medicine, Corvallis, OR, United States; ^6^ Transformative Health and Wellness, Corvallis, OR, United States; ^7^ Laboratory of Clinical Immunology and Microbiology, The National Institute of Allergy and Infectious Diseases (NIAID), The National Institutes of Health (NIH), Bethesda, MD, United States

**Keywords:** skin granuloma, vaccine-derived rubella virus, inborn errors of immunity, gene therapy, viral persistence, live virus shedding

## Abstract

A young man with X-linked severe combined immunodeficiency developed a persistent vaccine-derived rubella virus (VDRV) infection, with the emergence of cutaneous granulomas more than fifteen years after receipt of two doses of measles-mumps-rubella (MMR) vaccine. Following nasopharyngeal swab (NP) collection, VDRV was detected by real-time polymerase chain reaction (RT-qPCR) and sequencing, and live, replication-competent VDRV was isolated in cell culture. To assess duration and intensity of viral shedding, sequential respiratory samples, one cerebrospinal fluid sample, and two urine samples were collected over 15 months, and VDRV RNA was detected in all samples by RT-qPCR. Live VDRV was cultured from nine of the eleven respiratory specimens and from one urine specimen. To our knowledge, this was the first reported instance of VDRV cultured from respiratory specimens or from urine. To assess potential transmission to close contacts, NP specimens and sera were collected from all household contacts, all of whom were immunocompetent and previously vaccinated with MMR. VDRV RNA was not detected in any NP swabs from the contacts, nor did serologic investigations suggest VDRV transmission to any contacts. This report highlights the need to understand the prevalence and duration of VDRV shedding in granuloma patients and to estimate the risk of VDRV transmission to immune and non-immune contacts.

## Introduction

At least 300,000 people have been diagnosed with primary immunodeficiencies, or inborn errors of immunity, in the United States in 2018 (1). Among children with inborn errors of immunity, emergence of granulomas has been associated with rubella virus (RuV) (2, 3). Although vaccination against rubella is contraindicated for those with inborn errors of immunity, delays in immunodeficiency diagnosis can result in MMR vaccine being administered to these children. Additionally, the prevalence, persistence, and transmissibility of vaccine-derived rubella virus (VDRV) infection have yet to be fully elucidated. In this report, we describe a patient with late-diagnosis X-linked severe combined immunodeficiency (SCID-X1) and VDRV-associated granulomas and an assessment of VDRV shedding and potential transmission to the patient’s close contacts.

## Case description

**Figure 1:**
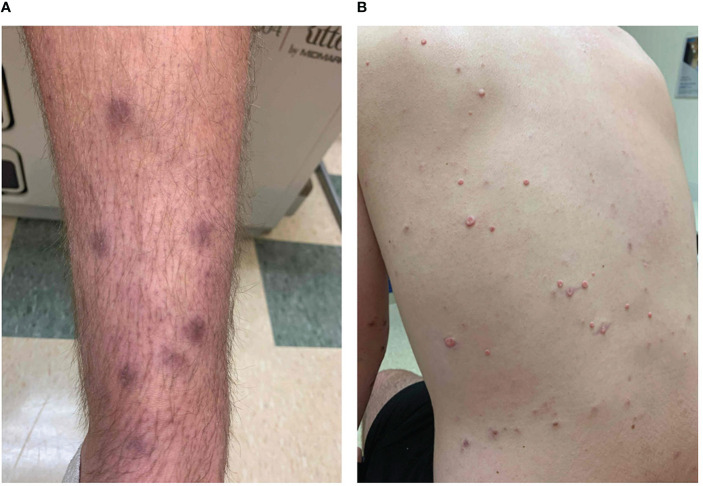
Clinical photographs. During dermatology consult, the patient reported that the diffuse hyperpigmented macular appearing lesions had erupted in January 2020 **(A)**, and additional, smaller lesions appeared on the torso by June 2020 **(B)**.

A 20-years-old man presented with a new rash to his primary care provider in June 2020. He had been previously diagnosed with SCID-X1 due to hypomorphic interleukin-2 receptor subunit gamma-chain (IL2RG) mutation in 2017, approximately 3 years prior to the onset of his rash. His immunologic evaluation was undertaken due to a history of severe eczema and frequent infections as well as a family history of immunodeficiency. Immunologic work-up was initially notable for hypogammaglobulinemia and he was placed on standard monthly intravenous immunoglobulin (IVIG) infusions. Genetic testing then returned positive for the c.460C>T (p.P154S) mutation in the IL2RG gene. At the time of presentation, the patient reported a six-month history of hyperpigmented, violaceous macules and plaques involving the extremities and torso but sparing the palms, soles, and mucous membranes (**Figure 1**). His skin lesions began on both feet and spread to the legs, torso, arms, and face over the course of a few months. At the time of initial evaluation, Tinea corporis was confirmed by potassium hydroxide preparation of a skin scraping, and flesh-colored papules with central umbilication clinically resembling those of molluscum contagiosum were also noted. Review of systems was negative for fevers, chills, arthralgias, respiratory, gastrointestinal, or genitourinary symptoms. Laboratory tests obtained eight months after appearance of the skin lesions were notable for a normal serum immunoglobulin G of 798 mg/dL and low CD4^+^ and CD8^+^ T-cell counts at 199 and <45 cells/µL, respectively. Very unusual for a SCID-X1 patient, he had above upper normal range numbers of CD3/CD16^+^56^+^ natural killer cells (1909 cells/µL, normal range 126-729 cells/µL). Prior to SCID-X1 diagnosis, he received routine childhood immunizations, including two doses of MMR vaccine at 1 and 4 years of age and a varicella vaccine. The patient reported two previous episodes of herpes zoster, persistent molluscum contagiosum, bacterial skin and soft-tissue infections, Tinea corporis, onychomycosis, and several episodes of viral and bacterial pneumonia. Prior to the onset of the skin lesions, he had no known exposure to tuberculosis or animal exposures. At the time of presentation, he was enrolled as a university student and lived with two roommates. During university breaks, he lived with his parents and a younger sibling, hereinafter referred to as close contacts.

**Figure 2:**
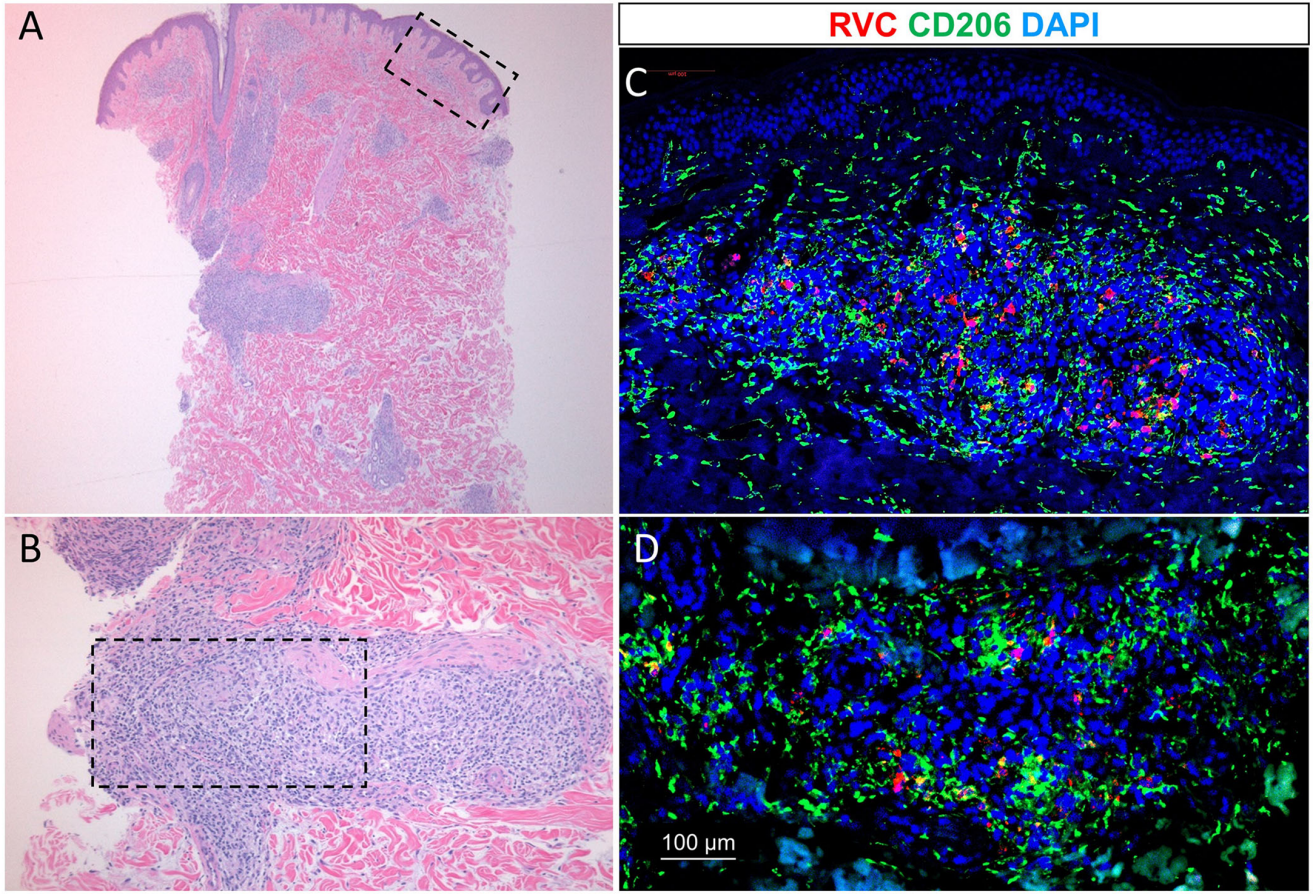
Low power [2.5x objective, **(A)**] and high power [10x objective, **(B)**] histologic examination of diffused skin granulomas (hematoxylin-eosin staining). Immunofluorescence staining of the granulomas with RuV anti-capsid antibody (red) in M2-macrophages (green) **(C, D)**. Blue nuclei were the result of counterstaining with 4’,6-diamidino-2-phenylindole (DAPI).

The patient underwent biopsy of a violaceous macular lesion on his left flank. Dermatopathology revealed superficial and deep dermal infiltrates, predominantly histocytes, aggregating into poorly formed granulomas centered around vessels and adnexal structures. The Gram, Periodic Acid-Schiff, fluorescent rhodamine acid-fast, Fite, and Grocott methenamine silver stains were negative, as were fungal and mycobacterial cultures. Double fluorescent immunohistochemical (IHC) staining of formalin-fixed paraffin-embedded (FFPE) tissue sections of the skin biopsy with mouse monoclonal antibody against RuV capsid and rabbit polyclonal antibody against CD206 (3, 4) revealed the presence of RuV in M2 macrophages in the cores of granulomas located in the upper (**Figures 2A, C**) and deep dermis (**Figures 2B, D**). No staining for RuV capsid was detected in a few myeloperoxidase (MPO)-positive neutrophils near the granulomas (not shown). IHC staining for measles virus and varicella zoster virus (VZV) antigens were negative (not shown).

### Virus characterization

**Figure 3:**
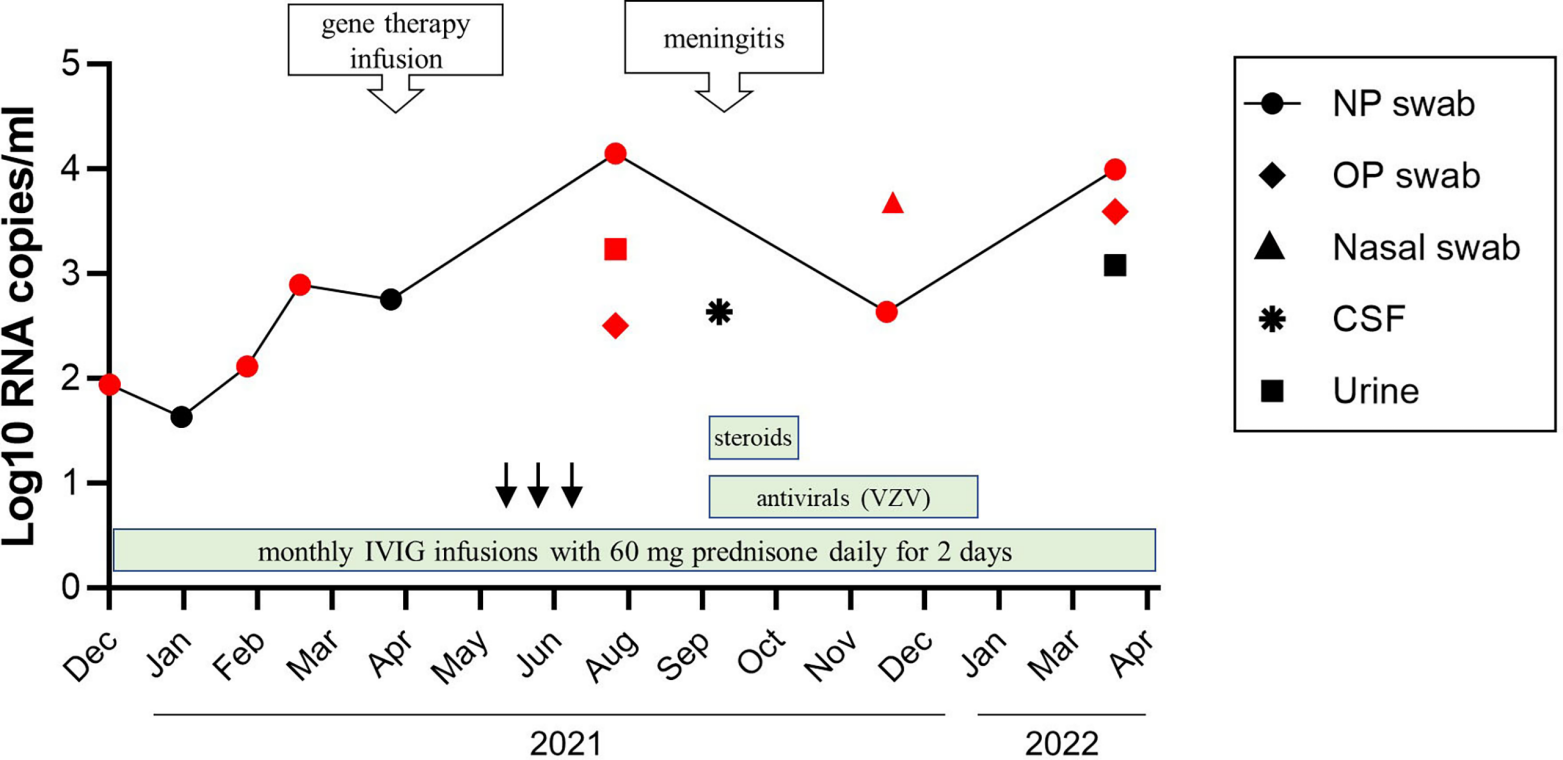
A timeline of viral shedding and treatments. Changes in VDRV RNA load in sequential patient’s samples were determined by RuV RT-qPCR. Samples positive by cell culture were indicated by the red color. Treatments are indicated by the green bars. The anti–VZV treatments included IV acyclovir 750 mg every 8 hours for 10 days followed by oral valacyclovir 1 g every 8 hours for 10 days, then oral acyclovir 800 mg twice daily after this as prophylaxis. Steroid treatments included IV hydrocortisone for 5 days (the first dose 75 mg, and then 50 mg doses), and one dose of IV methylprednisolone 80 mg given with IVIG. Treatments with colony stimulating factor were indicated by the black arrows.

Following consultation with an infectious disease specialist, an NP swab and fresh-frozen skin biopsy sample were collected for the detection of RuV RNA by RT-qPCR (5) in December 2020. RuV RNA was not detected in the skin biopsy sample. The failure to detect RuV RNA by RT-PCR in the fresh-frozen skin biopsy despite detecting RuV capsid by IHC in FFPE tissue sections is most likely due to the absence of RuV-positive cells in the frozen biopsy, as RuV is known to be unevenly distributed in lesions (4). However, RuV RNA was detected in the NP swab, and replication-competent RuV was isolated in Vero cell culture from the NP swab (**Figure 3**). Because the patient had no known recent exposures to persons with suspected or confirmed rubella, there was concern that the RuV had been acquired earlier, potentially through MMR vaccination (6). The complete genomic sequence of the isolated virus was determined using the Sanger method (7) and designated RVi/Oregon.USA/49.20 (GenBank accession number ON861827). Phylogenetic analysis showed that the isolated virus was vaccine-derived (**Figure S1**) with 97.3% nucleotide identity to the genomic sequence of the RA27/3 strain used in an MMR vaccine. A 36-nucleotide deletion in RVi/Oregon.USA/49.20 resulted in the N-terminal deletion of 12 amino acids of the E2 glycoprotein. Despite this deletion, the virus replicated efficiently in Vero cells, producing titers around 10^6^ focus forming units (ffu) per mL of the culture medium, which is 1–2 logs higher than those produced by other previously described VDRV isolates (7). Following these results, it was recommended that the patient adhere to droplet precautions until further information regarding potential VDRV shedding could be ascertained.

*Treatment.* No specific treatment is currently indicated for rubella virus infection (6, 8). The progression of the rubella virus infection and waning immunity, as indicated by a decline in CD4^+^ and CD8^+^ T-cell numbers in the absence of available anti-viral therapy for rubella, provided the rationale for the patient to undergo ex vivo lentiviral gene therapy at the National Institutes of Health (NCT01306019) with busulfan (6 mg/kg total) myeloid conditioning in April 2021 (9, 10). In September 2021, approximately 5 months after gene therapy, the patient was admitted for facial rash and meningitis. A swab collected from a facial skin lesion was positive for VZV by PCR. Cerebrospinal fluid (CSF) was positive for VZV as well as for VDRV by RT-qPCR. VDRV viral load was 4.3x10^2^ RNA copies/mL CSF; live VDRV was not recovered from CSF (**Figure 3**). He received IV acyclovir 10 mg/kg every 8 hours for 10 days and IV hydrocortisone 50 mg daily for 3 days for VZV treatment during his hospital admission. He was discharged home on oral valacyclovir 1 g every 8 hours for 11 days to complete a total 21-day course of antiviral therapy, then transitioned to acyclovir prophylaxis.

### Follow-up and outcomes

At time of report, patient was 9 months post gene therapy and pancytopenic with absolute neutrophil counts in the range of approximately 500-1000 cells/µL. Reconstitution of T-cells in older X-SCID patients takes years and at this early time point, his T-cell counts remained low with a CD4 count of 65 cells/µL (21%) and CD8 count of <45 cells/µL (4.1%). However, his violaceous skin lesions had mostly resolved by 9 months following gene therapy.

**Figure 4:**
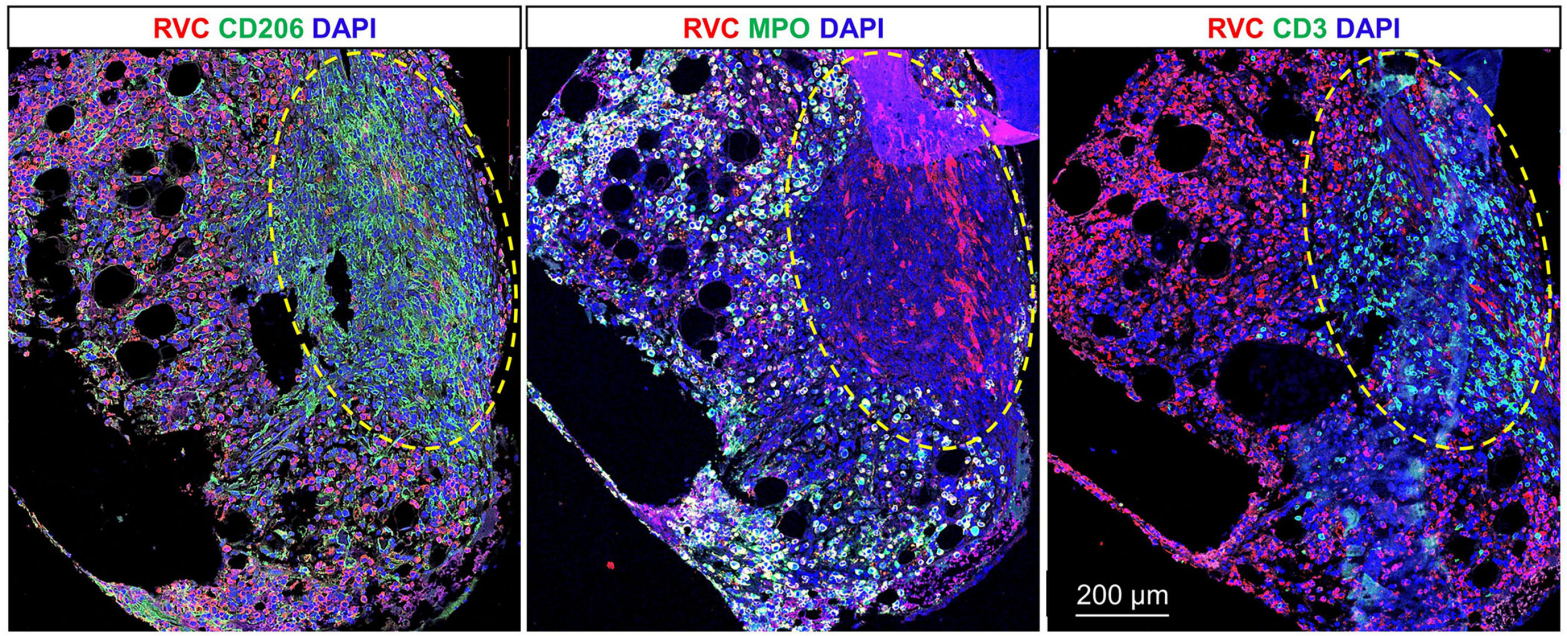
RuV-associated granuloma in bone marrow. Double immunofluorescent IHC staining of sequential tissue sections of bone marrow core biopsy for rubella capsid (RVC, red) and one of the cell type markers (green), CD206 (M2 macrophages), MPO (neutrophils), or CD3 (T cells) shows predominant rubella staining of neutrophils and less frequent rubella staining of M2 macrophages. Numerous RVC^-^CD3^+^ T cells were located inside the granuloma outlined with the yellow dashed line. Scale bar: 200 µm.

A bone marrow biopsy performed in July 2022 to evaluate ongoing pancytopenia (WBC 1.34 K/µL, hemoglobin 9.6 g/dL, and platelet count 20 K/µL) was notable for the presence of multiple foci of granulomatous inflammation. Grocott methenamine silver and acid-fast bacilli stains did not detect fungal and mycobacterial organisms in the bone marrow biopsy. There was no evidence of Epstein-Barr virus on *in-situ* hybridization. Qualitative PCR for cytomegalovirus, Epstein-Barr virus, human herpesvirus 6, and adenovirus was negative. To evaluate the possibility of VRDV as a possible etiology of the patient’s pancytopenia, the sequential tissue sections of bone marrow core biopsy were double immunostained with RuV capsid antibody and either CD206, MPO, or CD3 antibody (**Figure 4**). The bone marrow was strongly positive for rubella capsid with MPO^+^ neutrophils being the main infected cells and almost all neutrophils were rubella-positive. There was heavy infiltration of CD206^+^ macrophages, many of them forming granulomas. Numerous CD3^+^ T cells were predominately localized inside the granulomas. The areas occupied by granulomas were free from rubella-positive neutrophils, but some macrophages inside the granulomas contained rubella antigen. In addition, macrophages with rubella-positive neutrophils or globules of rubella antigen inside the cells could be seen throughout the sample outside of granulomas. The lack of IHC staining of neutrophils and macrophages with VZV monoclonal antibody cocktail confirmed the specificity of rubella antibody staining of these cells (not shown).

### Assessment of viral shedding

To monitor virus shedding by this patient, 11 sequential respiratory samples (eight NP swabs, one nasal swab, two oropharyngeal swabs) and two urine samples were collected over 15 months beginning in December 2020. The samples were analyzed by quantitative RT-qPCR and viral cell culture. VDRV RNA was detected in all eight NP swabs; replication-competent viruses were recovered from six of these (**Figure 3**). The amount of virus in the NP swabs varied between 4.3x10^1^ to 4.3x10^2^ RNA copies/mL of swab media with two spikes of RuV RNA concentration. One spike of 1.4x10^4^ RNA copies/mL was observed three months after gene therapy in July 2021, and the second spike of 9.9x10^3^ RNA copies/mL occurred in March 2022, four months after treatment with steroids. IVIG had been administered monthly during this period. Two oropharyngeal swabs, a nasal swab, and urine collected in July 2021, contained replication-competent VDRV as well. Only one urine sample and two NP swab samples were culture-negative, and each was collected at a different time point. Viral RNA loads in the culture-negative samples were compatible to those in the culture-positive samples.

### Transmission assessment

Though VDRVs have been detected in some individuals with inborn errors of immunity by culture in skin lesions and by RT-PCR in NP swabs (4, 7), and RuV vaccine has been hypothesized to be an antigenic trigger for development of cutaneous granulomas (2, 3), transmissibility of VDRVs remains unknown. To assess potential transmission of VDRV from the patient to his close contacts, NP swabs and serum samples were collected from three close contacts in the family, including an immunocompetent adolescent sibling. NP swabs were tested by rubella RT-qPCR. Serologic investigation included measuring immunoglobulin M (IgM) and IgG to rubella virus, and 50% neutralization titer (NT_50_) against the RA27/3 rubella virus strain as previously described (7). The patient’s rubella serologic status was also assessed. The NP swabs of these close contacts were negative by RT-qPCR. None of the serum samples contained rubella IgM. IgG titers of the close contacts ranged from 25–60 IU/mL, and the NT_50_ was 80 (**Supplementary Table 1**); both results were similar to those of typical immunocompetent vaccinees (7). In contrast, the serum neutralization titer of the patient was substantially higher (NT_50_ = 640) than that of his close contacts or other immunocompetent vaccinees. Laboratory testing did not provide evidence of VDRV transmission to close contacts, including a younger sibling who could be potentially exposed prior to receipt of MMR vaccination, suggesting that VDRV is unlikely to have been transmitted to vaccinated contacts with competent immune systems. In light of these results, the infectious disease physician and public health officials advised the patient that the droplet precautions suggested earlier for infection control could be discontinued.

## Discussion

To our knowledge, this is the first reported detection of shedding of live, replication-competent VDRV into respiratory secretions or urine. Previously, shedding of VDRV RNA into NP secretions has been reported, but no live virus has been recovered in cell culture (7, 11, 12). Granulomatous skin lesions were the only sites from which live VDRVs were isolated previously (7, 12). Moreover, this is the first reported case of continuous shedding of live VDRV for at least 15 months. The patient’s virus is highly divergent from the vaccine virus and replicates more efficiently than the VDRV strains recovered from skin lesions (7), but its teratogenic potential is not possible to estimate at present, given the lack of a suitable animal model for teratogenesis caused by rubella virus.

RuV capsid antigen has been detected in this case and previously in M2 macrophages in multiple rubella-associated M-type granulomas in dermis and hypodermis, and in bone marrow neutrophils and granulomas (3, 4, 13). However, the structure and composition of bone marrow granuloma in this case were different from the previously described granuloma in bone marrow of patient P4 (4). The granulomas in P4 case were poorly formed and consisted mainly of rubella positive macrophages, while the well-formed M-type granulomas in this case contained abundant T cells intermixed with predominantly rubella-negative CD206^+^ macrophages. The presence of T cells in the areas cleared from rubella antigen suggests the restoration of T-cell effector functions after the gene therapy that resulted in regression of the skin lesions. Although skin lesions have been almost resolved after gene therapy treatment, shedding of the live virus continued and pancytopenia persisted. The likely source of the persistently shed virus is VDRV-infected neutrophils still present in the bone marrow 15 months after gene therapy treatment. Pancytopenia may be a result of ongoing inflammation in the bone marrow as VDRV infection and pro-inflammatory cytokines produced by activated M2 macrophages and T cells can interfere with hematopoiesis (14). In addition, the absence of neutrophils in the skin lesions, persistent cytopenia, and the abundance of rubella-positive neutrophils in the bone marrow, taken together, suggest that RuV infection may interfere with maturation and subsequent egress of the infected neutrophils from the bone marrow. This patient also raises some very interesting scientific questions about the role of natural killer cells and neutrophils in rubella- associated chronic inflammation and other aspects of RuV associated granulomatous disease. Since this patient (and other similar patients) are usually undergoing various treatments for the disease, many of these questions need to await accumulation of information to justify more detailed clinical studies.

IgG antibody in the patient’s serum most likely originated from IVIG infusions; thus, his IgG titer was consistent with that of immunocompetent vaccinees as well. High neutralization titer against RA27/3 is a characteristic feature of patients with VDRV persistence (7), including immunocompetent adults (10). Given that the case patient had been vaccinated with MMR more than fifteen years before the granulomas appeared and prior to SCID-X1 diagnosis, it is likely that the case had persistent subclinical VDRV infection, and that the viable VDRV had emerged concurrent to the appearance of VDRV-affected lesions. Encouragingly, despite prolonged close contact with family members, there was no indication of transmission to them, as might be evidenced by the presence of VDRV in NP secretions or elevated RuV antibody titers. It should be noted, however, that the patient did not live at home during the period of documented VDRV shedding and stayed with family only intermittently during short visits and university breaks and that his household contacts were immunocompetent and vaccinated. Unfortunately, it is not possible to determine the onset or duration of VDRV shedding in the patient prior to this study. Limited knowledge of the markers of subclinical rubella infection and rubella re-infection and their unique expression and timing is another limitation of the study, which hampers investigations of VDRV transmission to vaccinated individuals.

## Conclusion

This report highlights the strength of a multidisciplinary team approach to diagnosing and treating rubella-associated pathologies. This is a rare case, and only additional investigations can identify whether other immunocompromised individuals persistently shed live VDRV. Therefore, the frequency of live VDRV shedding and potential VDRV transmissibility to immune and especially non-immune individuals should be further investigated in prospective studies to determine the risk, if any, posed to the maintenance of the current rubella and congenital rubella syndrome elimination status in the United States. Though the absence of transmission to close, vaccinated contacts in the current setting is reassuring, we cannot rule out the possibility that VDRV might be transmitted from such a patient to unvaccinated pregnant people, their fetuses, and infants 6–12 months of age, after waning of maternally acquired antibody but prior to receipt of MMR, as reported elsewhere (15). Although exposures are likely rare, the development of a public health strategy for VDRV should be considered to mitigate VDRV transmission to those at elevated risk of infection.

## Patient perspective

Being diagnosed with a rare condition such as X-SCID can be scary and overwhelming, but it has also given me the opportunity to help the immunology community learn more about my condition. This has been a rewarding silver lining of my diagnosis because the valuable information that the CDC and the NIH are gathering from my case could help to streamline treatments for future patients. This knowledge has helped me to deal with the extra testing and procedures involved with being a participant in these studies, as it is a small sacrifice if it can help improve future treatments for X-SCID.

## Data availability statement

The data that support the findings of this study are available from the corresponding author upon reasonable request. The data are not publicly available due to Danish legislations.

## Ethics statement

Written informed consent was obtained from the individual(s) for the publication of any potentially identifiable images or data included in this article.

## Author contributions

LP, KB, PC, RL, MS, TL, and ES contributed to conception and design of the study. ES, JL, MS, ED, CL, RL, SD contributed patient’s clinical data. LP, RF, MC, and LH contributed to analysis of patient’s samples and data interpretation. Drafting of the manuscript: KB, RL, LP, and PC. Critical revision of the manuscript: all authors. All authors contributed to the article and approved the submitted version.
