# Supplementary material for: Case report: Persistent shedding of a live vaccine-derived rubella virus in a young man with severe combined immunodeficiency and cutaneous granuloma
Source: Front Immunol. 2022 Dec 8;13:1075351. doi: 10.3389/fimmu.2022.1075351 (PMC9773200; doi:10.3389/fimmu.2022.1075351)
Supplement: Supplementary file 1 [file DataSheet_1.pdf]

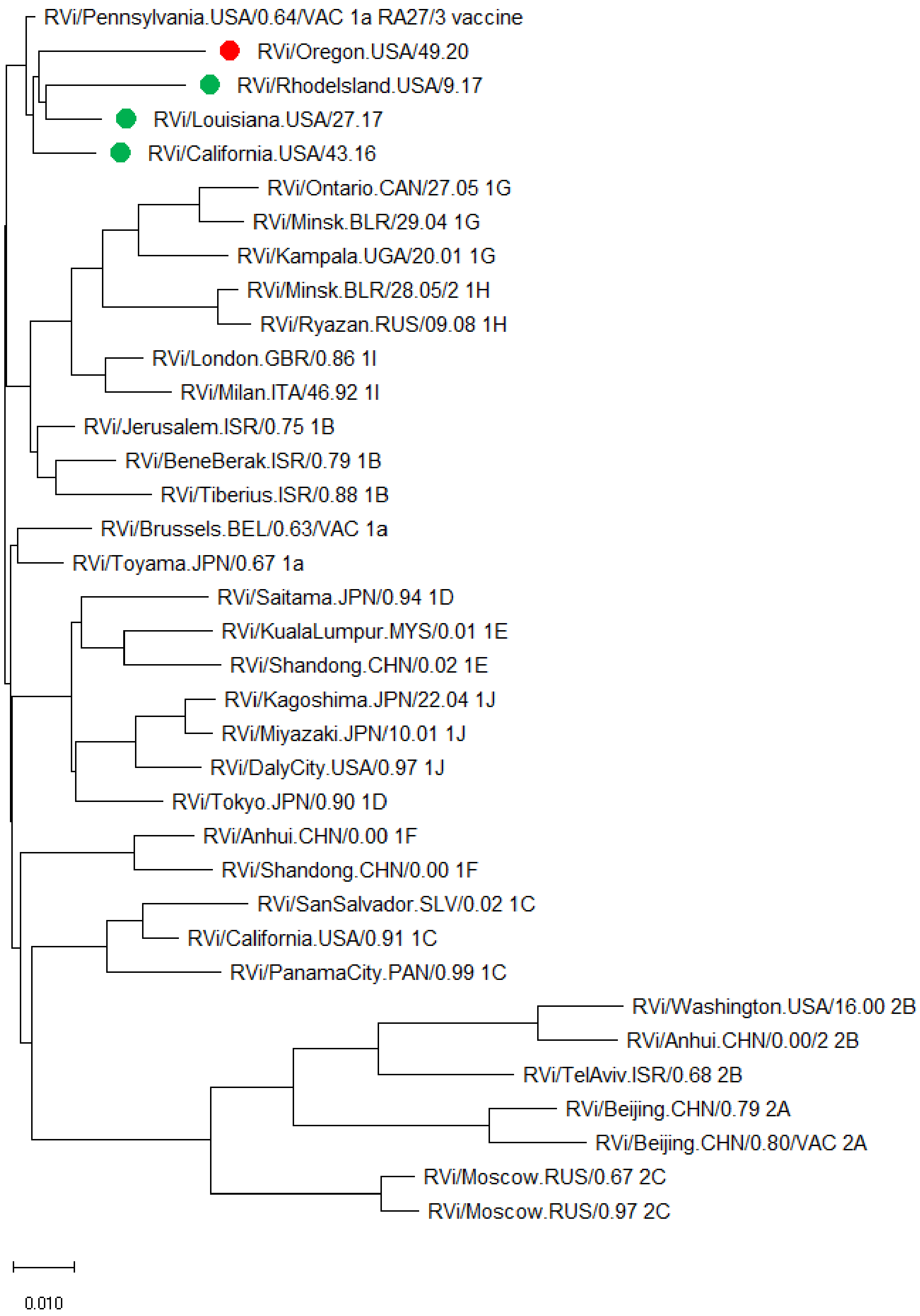

Supplementary Figure 1. Phylogenetic tree shows the genetic relationships between the whole genome sequences of the isolate from the NP swab of the current patient (red dot), iVDRV isolates from the skin biopsies of the three previously described patients (green dots), RA27/3 vaccine, and the 32 WHO reference viruses. The tree was constructed using the Maximum Likelihood method in MEGA10. All taxa are labeled with WHO names. The scale bar indicates the number of base substitutions per site. RA27/3, the patient's virus, and iVDRVs represent a separate branch on the tree with RA27/3 being basal.
