# Supplementary material for: Case report: Persistent shedding of a live vaccine-derived rubella virus in a young man with severe combined immunodeficiency and cutaneous granuloma
Source: Front Immunol. 2022 Dec 8;13:1075351. doi: 10.3389/fimmu.2022.1075351 (PMC9773200; doi:10.3389/fimmu.2022.1075351)
Supplement: Supplementary file 2 [file Table_1.docx]

**Supplementary Table 1.** Results of RuV RT-PCR and serologic assays for the patient and close contacts

|  | **RuV serology** | | | | **RuV RT-PCR** | | |
| --- | --- | --- | --- | --- | --- | --- | --- |
|  | **Sample** | **IgM** | **IgG, IU/mL** | **NT_50_** | **Sample** | **Ct** | **Result** |
| Patient | serum | Negative | 111 | 640 | NP | 29 | Positive |
| CC1 | serum | Negative | 25 | 80 | NP | >40 | Negative |
| CC2 | serum | Negative | 39 | 80 | NP | >40 | Negative |
| CC3 | serum | Negative | 60 | 80 | NP | >40 | Negative |

RuV: rubella virus; CC: close contact; NP: nasopharyngeal swab; Ct: Cycle Threshold; IgG: Immunoglobulin G; IgM: Immunoglobulin M; NT_50_: The highest dilution of the serum at which the input virus signal was reduced by at least 50% within the dilution series; IU/mL: International Units per milliliter**.**
